# Correlation between two-point discrimination with other measures of sensory loss in diabetes mellitus patients

**DOI:** 10.4103/0973-3930.44076

**Published:** 2008

**Authors:** R. Periyasamy, M. Manivannan, V. B. Narayanamurthy

**Affiliations:** Biomedical Engineering Group, Department of Applied Mechanics, Indian Institute of Technology Madras, Chennai - 600 036, India; 1 Diabetic Foot Clinic, Sundaram Medical Foundation, Chennai - 600040, India

**Keywords:** Diabetic neuropathy, pedopowergraph, sensory measures, shoremeter

## Abstract

Diabetic neuropathy is one of the most important factors for foot ulceration in diabetes mellitus (DM) patients. Among different sensibility measures of neuropathy, two-point discrimination (TPD) has been suggested as a reliable method; however, the correlation of TPD with other well-known measures is not known. We measured the loss of protective sensation using Semmes-Weinstein Monofilaments (SWMF), hardness of the foot sole using shore meter (sh), power ratio (PR) using pedopowergraph and TPD using esthesiometer in foot areas of both left and right legs in 14 DM subjects. We have found no correlation either between TPD and shore values (sh) or between TPD and PR. The SWMF (10 g) is found not to provide any additional value in measuring loss of sensation in comparison to TPD. The TPD appears to be measuring different property of the foot compared to other measures. The mechanism of this independence is not well understood and more investigation is required to understand the mechanism.

## Introduction

Diabetic neuropathy is the primary complication and most likely the cause of morbidity and mortality related to diabetes. It is one of the most important factors for foot ulceration in diabetes mellitus (DM) patients. Prevention of these problems is difficult, mainly because there is no method to correctly access sensibility of the foot. Evaluation of sensibility on the feet of diabetic patients is an important factor in order to prevent ulceration at risk. Various modalities of touch sensation like pressure, vibration and two-point discrimination (TPD) are used to test sensation loss or sensibility. Generally, sensibility is defined as normal touch, diminished light touch, diminished protective sensation and loss of protective sensation.[1] Loss of protective sensation is measured using[2,3] a Semmes-Weinstein monofilament (SWMF), which exerts 10 g force when pressed perpendicularly against the skin of the foot sole at risk. SWMF broadly classifies people into two large groups, one who have and other who have no sensation of 10 g SWMF. The latter group is at risk of ulceration.[4] SWMF is less useful in screening the loss of protective sensation beyond the risk factor.[5]

Boulton *et al*,[4] Patil *et al*[6] and Prabhu *et al*,[7] have defined peak foot pressures and shown that the loss of protective sensation is the cause for plantar ulcers in diabetic neuropathy.[7] The latter quantified loss of sensation into three levels[1] and reported good correlations between quantified levels of sensation loss and peak foot pressure. They used the actual foot pressure distribution to calculate a new parameter power ratio (PR). PR is the ratio of high-frequency power to the total power in the foot pressure image distribution related to the three levels of sensation loss of foot sole in diabetic neuropathy. Romanelli *et al*,[8] have proposed ‘shoremeter’ for measuring the hardness of soft materials like rubber and skin. Piaggessi *et al*,[9] have measured hardness of the foot sole using ‘durometer’.

The measurement of the cutaneous sensation to differentiate one-point from two-point static touch stimuli may allow identification of ulcer earlier in the clinical course of diabetic neuropathy.[10] TPD has been suggested as a reliable quantitative measure of sensibility.[11] TPD, static and moving, has been used as a tool to measure sensory loss in DM. Although the method is subjective, as the patient must report whether or not the pressure is felt, it is more reliable than the previously available methods and it is a quantitative measure of the sensory loss.

Although TPD is more reliable, little work is done comparing it with other measures of sensibility for diabetic patients. The shore level[12] increased from 15 to 40 corresponding to an increase in loss of sensation level from 3 g to 10 g of SWMF with very good correlation between these variables. Charanya *et al*,[12] observed that the increase in mean PR for an increase in shore value of foot sole soft tissue from 20 to 25 was of the order of four times the corresponding increase for a change in shore values from 10 to 15 in the same foot sole areas. Correlation of TPD with other well-known measures is not known in literature. Dellon *et al*,[13] observed that there is no correlation between TPD and shore values in fingers, not in lower extremities which is affected first in diabetic patients. Knowing these correlations in diabetic patients could help us to avoid duplication of clinical tests and reduce overall healthcare cost.

In this paper, we present our study on the correlation of TPD with other measures such as loss of protective sensation, hardness of the sole and PR in foot areas of DM patients. We found that there is no correlation between TPD and shore value in the feet, similar to the observation by Dellon *et al*,[13] in fingers and no correlation between TPD and PR value in the feet. As shore values and PR were found to have a high correlation, TPD could represent advanced and reliable measure to predict the gradual loss of sensation in DM patients.

### Two-point discrimination

The TPD test was originally used for innervations density test of afferent fibers.[14] Discriminating the two anatomical points by varying the distance between the two prong points measures the degree of sensation loss and detects progressive loss of sensation in the foot. Among the two types of TPD, static TPD (STPD) and dynamic TPD (DTPD), the former is commonly used in emergency departments to determine digital nerve integrity.[15] It is the current recommended method for physicians evaluating loss of sensation or degree of sensation loss in diabetic patients. DTPD is usually measured with disk-criminator,[15] moving the prongs along the surface of the center. Though STPD and DPTD have been used as tools to measure sensory loss in DM, the DTPD values were of lesser magnitude than STPD values in all anatomical areas tested. The DTPD is not routinely used in clinical practices.

Calipers or an opened paper clip with two parallel ends are used for finding STPD.[16] Esthesiometer, a modified form of vernier calliper, is clinically used for determining the TPD of touch, by moving the prongs into contact with the portion of the body part and then pressing until the patient feels a sensation. A set of small grating surfaces was recently introduced for cutaneous spatial resolution measurement. The gratings are placed on the skin and subjects are required to identify the orientation of grooves and bars. The finest grating whose orientations are discriminated reliably provides an estimate of the spatial resolution limit in the tested area.[17] In the 1990s, Dellon proposed the pressure-specified sensory device (PSSD) that could gather information about static and moving touch in a continuous form by using a computer.[11] PSSD is a quantitative sensory device, which consists of one or two blunt probes and sensitive transducers to measure and record the perception thresholds of pressure on the surface of the body in g/sq mm.

## Methodology

We measured the loss of protective sensation, hardness of the sole, PR and (14 diabetic subject, 4 normal subjects). TPD in foot areas of both left and right legs of 18 subjects. Details of diabetic subjects are shown in Table 1. Measures from the normal subjects closely match with that of literature data.

**Table 1:** Details of diabetic subjects

Diabetic subjects		Age (years)	No. of subjects with callosity	Duration of diabetes mellitus (years)*
Male	Female	50–70	3	
5	9			5-20

* for both men and women

If the subject had callosite in any of the foot area, the TPD and other measures were taken adjacent to the callosite, but within the same area of the foot. The subject's age, duration of the DM and medication were noted, but were not considered for our analysis.

Study period was from January to March 2007. A total of 18 subjects were tested and the details of each diabetic subject are given above in the Table 1 All the subjects were clinically screened for peripheral artery occlusive disease (PAOD). There were no subjects with clinical vasculopathy.

In order to simplify our analysis, we divided the foot region into different areas.

**Figure 1:**
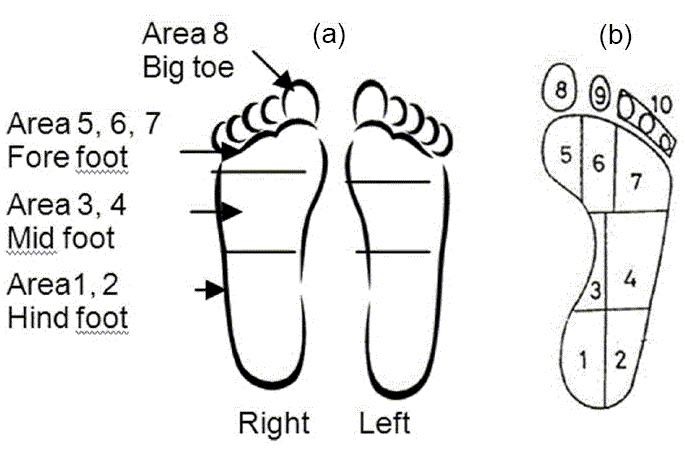
(a) standard division of foot area, (b) division of foot for our study

In the literature, the foot is divided into ten standard significant areas [Figure 1 (a)], as per method indicated in Cavanagh *et al*,[18] and Patil *et al.*[19] For our analysis, we have divided each foot into four areas as mentioned in Figure 1(b). Hind foot includes area 1 and 2; mid foot includes area 3 and 4; fore foot includes area 5, 6 and 7; and the big toe is area 8.

### Loss of protective sensation measurement using SWMF

Each footprint is divided into four standard areas as per the method indicated [Figure 1(b)]. To test the sensation, the patient sits with the eyes closed and the foot being placed in a comfortable position. The SWMF (10 g) is pressed perpendicular to the surface of the foot sole with a force just sufficient to buckle the monofilament to find the sensation level. The patient is asked to say ‘yes’ when he/she feels the monofilament. Then the detected sensation level is noted in the datasheet. The above procedure was done for 14 diabetic subjects and four normal subjects in the prescribed locations as shown in the Figure 1(b).

### Foot sole hardness measurement using SM

For measuring the hardness of the foot sole, the patient's foot is placed in a comfortable position and the shoremeter is pressed perpendicular to the surface of the foot sole and the depth of an indentation, indicated by the pointer of the instrument, in the material created by a given force on a standardized pressure foot is noted on the datasheet. The shoremeter reads the hardness in degree shore values from 0 to 100. Three trials are performed at each site of foot sole and the average value noted. When shore levels are measured on foot sole of diabetic patients, they can be any combinations of 20–60 degrees shore in different foot sole areas depending upon the level of foot sole hardness and diabetic neuropathy. The above procedure was done for 14 diabetic subjects and 10 normal subjects in the prescribed locations as shown in Figure 1(b).

### Measurement of foot sole pressure using pedopowergraph

**Figure 2:**
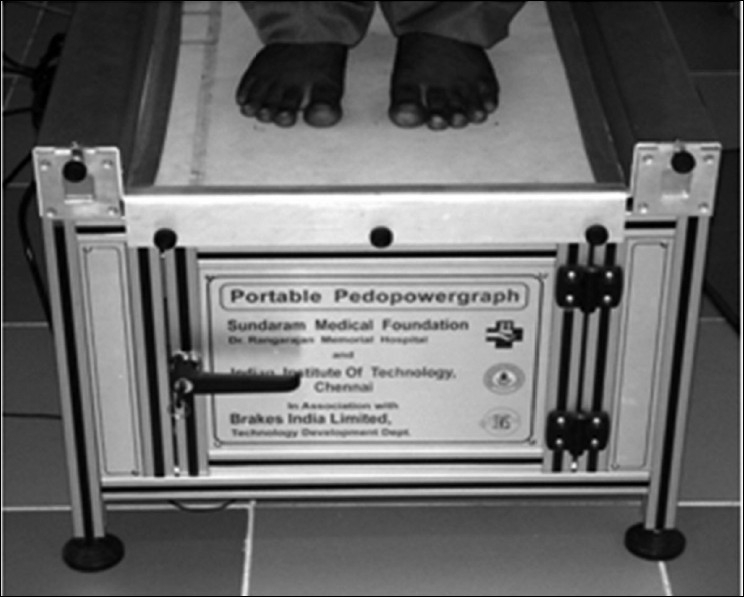
Pedopowergraph

We used pedopowergraph (PPG) system as mentioned.[19] It is based on continuous transduction principle and measures pressure distribution parameter PR. PR is defined as the ratio of high frequency power to the total power in the power spectrum of the standing foot pressure image obtained by PPG.[12] The patients are first asked to stand straight on the PPG machine as shown in Figure 2. A reference image and an image of the foot sole of diabetic patient are captured. The footprint images are divided into four areas (from heel to toes) and the number of samples, M and N, in a particular foot sole area depending on the size of the particular area of the foot and corresponding image size is represented as (M × N) pixels. The Fourier spectrum F (u, v) of an image, f (x, y) corresponding to a foot area is obtained using equation (1). The spatial frequencies (u and v) are denoted by cycles per distance and for this analysis, since the image size (distances) is given in terms of pixels, the spatial frequencies are represented by cycles per pixels

(1) F(u,v)=1MN∑x=0M−1∑y=0N−1f(x,y)e−j2πuxvy+MN

For u = 0, 1, 2,…M−1, v = 0, 1, 2,…N−1

Spatial frequencies and their distributions of these images are analyzed by performing the 2-D discrete fourier transform (DFT) using MATLAB version 6.1. Using the periodicity property of DFT,[20] the fourier spectrum is shifted to the center of frequency plane.

The DC component, F (0.0) is deleted as it gives only the average value of the image intensity. The magnitudes of the power spectrum, in each of the foot areas, are obtained by squaring the magnitudes of Fourier spectrum[2] of light intensity variations of foot images and the total power, TP in the image is obtained using equation (2).

(2) $$\text{TP}=\left\{\sum_{u=-\frac{M}{2}}^{\frac{M}{2}}\sum_{v=-\frac{N}{2}}^{\frac{N}{2}}{\left|F(u,v)\right|}^{2}\right\}-{\left|F(0,0)\right|}^{2}$$

Since for a foot area image, M and N are different, depending on the size of the particular area, the cut-off frequency D_0_ (cycles per pixel) that separates the lower and higher spatial frequency components is defined by equation (3).

(3) D0={M4N4if      N≥Mif      N≤M

Where D (u, v) is the distance from point (u, v) to the origin of the frequency plane given by equation (4) as follows

(4) $$D(u,v)=\sqrt{u^{2}+v^{2}}$$

The low frequency power (LFP) in Volt^2^ and high frequency power (HFP) in Volt^2^ are calculated using equations (5) and (6), respectively.

(5) LFP=∑D(u,v)=0D0F(u,v)2−F(0,0)2

(6) HFP=TP−LFP

Now, the PR is calculated by using equation (7) as follows. Multiplication by 100 is to express the PR value as a whole number.

(7) $$\mathrm{PR}=\left(\frac{\mathrm{HFP}}{\mathrm{TP}}\right)\times 100$$

The parameter, PR is evaluated for diabetic feet with different levels of loss of sensation and shore levels. The above procedure was done for 14 diabetic subjects and four normal subjects.

### TPD measurement using esthesiometer

The TPD thresholds were assessed using esthesiometer. The patients were first placed in a comfortable reclining position with eyes closed. Then esthesiometer was used to find the TPD. The two prong tips are made to touch the body part at the same instant. The subject orally stated whether he/she perceived the touch as a single point or as two separate points. Occasionally and without the subject's knowledge, the subject is touched with only one prong. This prevents the subject from knowing whether or not a double-point stimulus was always delivered. When the subjects consistently perceive one point rather than two points, the TPD is reached and this is recorded in the datasheet. The above procedure was done for 14 diabetic subjects and four normal subjects, respectively.

## Results

**Figure 3:**
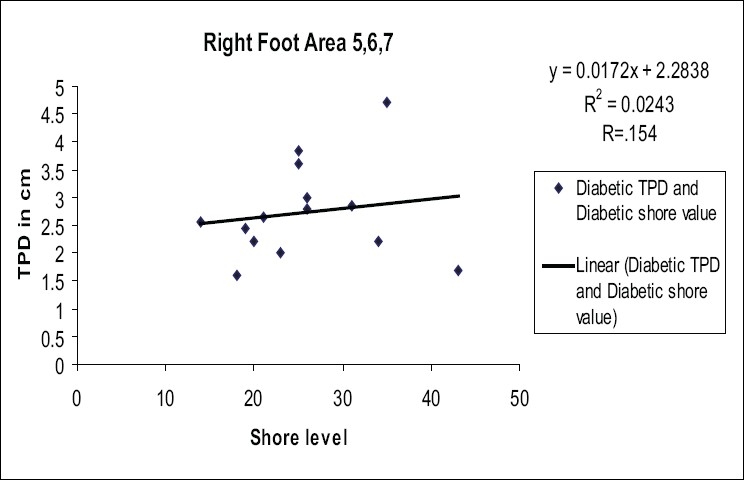
Variation of two-point discrimination with different shore level of foot sole in the right foot area 5, 6 and 7 for 14 diabetic subjects

**Figure 4:**
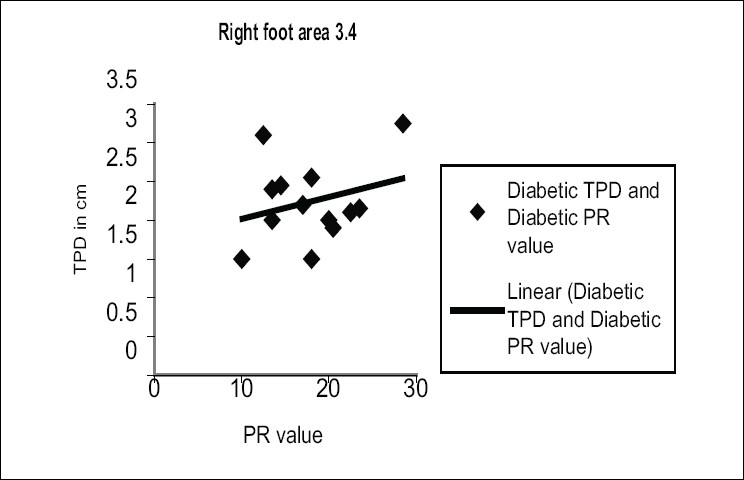
Variation of two-point discrimination with different PR value of foot sole in the right foot area 3 and 4 for 14 diabetic subjects

In order to present our results for diabetic subjects, we classified the subjects as ‘with sensation’ and ‘without sensation’. If the subjects could feel 10 g of SWMF in all areas of the foot, then they were classified as ‘with sensation’ and if they could not feel the monofilament in any of the foot area, they were classified as ‘without sensation’. We present our study for 14 DM subjects, among whom five subjects were ‘with sensation’ and nine subjects were ‘without sensation’. The following graphs show [Figures 3 and 4] the mean TPD values. The TPD measures are correlated with other measures such as shore and PR values, which is detailed in the following section.

### Correlation between diabetic foot two-point discrimination and diabetic foot sole (hardness) shore values

Figure 3 represents the variation of TPD with different shore levels of foot sole in the right foot area 5, 6 and 7 for 14 diabetic subjects. The correlation coefficient (*r*) was found to be 0.154. It shows that the two values measure different properties of the diabetic foot.

The above correlation has been calculated for each area and presented in Table 1. The corresponding regression equation was obtained by analysis of data of 14 diabetic subjects in all specified areas of the foot sole. It is clear from the Table 1 that all the areas do not have any correlation between shore level and TPD values. Therefore, higher levels of hardness of foot sole does not give rise to higher values of TPD.

### Correlation between diabetic foot two-point discrimination and diabetic foot sole (hardness) shore values – subjects without sensation

The correlation has been calculated for each foot area in ‘with sensation’ subjects and presented in Table 2. The corresponding regression equation was obtained by analysis of data of five diabetic subjects in all specified areas of the foot sole. It is clear from the Table 2 that some areas do not have any correlation between shore level and TPD values except in area 5, 6 and 7. Therefore, higher levels of hardness of foot sole does not give rise to higher values of TPD.

**Table 2:** Coefficient of correlation (r) between two-point discrimination and shore values (sh) and the corresponding regression equations in different foot areas of the foot in diabetic subjects with intact sensation

Foot areas	Correlation coefficient ( r )	Regression equation
Right 1, 2	0.745	TPD = −0.2262 Sh + 8.7659
Right 3, 4	0.296	TPD = −0.0238 Sh +2.7627
Right 5, 6, 7	0.64	TPD = 0.13 Sh +0.0028
Right 8	0.559	TPD = −0.0957 Sh +0.3429
Left 1,2	0.189	TPD = −0.0811 Sh +4.8434
Left 3,4	0.63	TPD = −0.1034 Sh +4.527
Left 5,6,7	0.987	TPD = 0.1954 Sh −1.8209
Left 8	0.00006	TPD = −0.0003 Sh 2.3767

### Correlation between diabetic foot two-point discrimination and diabetic foot sole (hardness) shore values – subjects without sensation

The correlation has been calculated for each area in ‘without sensation’ subjects and presented in Table 3. The corresponding regression equation was obtained for nine diabetic subjects in all specified areas of the foot sole. It is clear from the Table 3 that all the areas do not have any correlation between shore level and TPD values. Therefore, higher levels of hardness of foot sole do not give rise to higher values of TPD.

**Table 3:** Coefficient of correlation (r) between two-point discrimination and shore values (sh) and the corresponding regression equations in different foot areas of the foot in diabetic subjects without sensation

Foot areas	Correlation coefficient (r)	Regression equation
Right 1, 2	0.394	TPD = −0.108 Sh +6.4315
Right 3, 4	0.114	TPD = −0.02 Sh +2.9694
Right 5, 6, 7	0.044	TPD = −0.0056 Sh +2.974
Right 8	0.361	TPD = −0.0191 Sh +3.2338
Left 1, 2	0.170	TPD = -0.0288 Sh +3.761
Left 3, 4	0.614	TPD = 0.2793 Sh -4.7084
Left 5, 6, 7	0.00007	TPD = -0.0002 Sh +2.9608
Left 8	0.308	TPD = -0.0287 Sh +3.3106
Right 1, 2	0.356	TPD = −0.0836 Sh +5.4331
Right 3, 4	0.109	TPD = −0.0181 Sh +2.8107
Right 5, 6, 7	0.154	TPD = 0.0172 Sh +2.2838
Right 8	0.219	TPD = −0.0131 Sh +3.0161
Left 1, 2	0.118	TPD = −0.0217 Sh +3.4645
Left 3, 4	0.561	TPD = 0.1884 Sh −2.2079
Left 5, 6, 7	0.260	TPD = 0.0275 Sh +2.0772
Left 8	0.216	TPD = −0.0207 Sh +3.0011

### Correlation between diabetic foot two-point discrimination and diabetic foot power ratio values

Figure 4 represents the variation of TPD with different PR values of foot sole in the right foot area 3 and 4 for 14 diabetic subjects. The correlation coefficient (*r*) was found to be 0.227.

The above correlation has been calculated for each area and presented in Table 4. The corresponding regression equation was obtained by analysis of data of 14 diabetic subjects in all specified areas of the foot sole. It is clear from the Table 4 that all the areas do not have any correlation between PR and TPD values. Therefore, higher PR values of foot sole do not give rise to higher values of TPD.

**Table 4:** Coefficient of correlation (r) between two-point discrimination and Power ratio (PR) values and the corresponding regression equations in different foot areas of the foot in diabetic subjects

Foot areas	Correlation coefficient (r)	Regression equation
Right 1, 2	0.95	TPD = 0.363 PR −5.7705
Right 3, 4	0.063	TPD = −0.0048 PR +2.2371
Right 5, 6, 7	0.109	TPD = −0.0328 PR +3.2591
Right 8	NA	
Left 1, 2	0.309	TPD = −0.0715 PR +4.251
Left 3, 4	0.82	TPD = −0.0768 PR +3.536
Left 5, 6, 7	0.78	TPD = −0.1202 PR +5.358
Left 8	0.252	TPD = −0.035 PR +2.65

### Correlation between diabetic foot two-point discrimination and diabetic foot power ratio values – subjects with sensation

The above correlation has been calculated for each area in ‘with sensation’ subjects and presented in Table 5. The corresponding regression equation was obtained by analysis of data of five diabetic subjects in all specified areas of the foot sole. It is clear from the Table 5 that all the areas do not have any correlation between PR and TPD values. Therefore, higher PR value of foot sole does not give rise to higher values of TPD.

**Table 5:** Coefficient of correlation (r) between two-point discrimination and Power ratio (PR) values and the corresponding regression equations in different foot areas of the foot in with intact sensation diabetic subjects

Foot areas	Correlation coefficient (r)	Regression equation
Right 1, 2	0.158	TPD = 0.0482 PR +1.7733
Right 3, 4	0.227	TPD = 0.0282 PR +1.7344
Right 5, 6, 7	0.063	TPD = 0.008 PR +2.4053
Right 8	0.1	TPD = 0.0165 PR +2.4667
Left 1, 2	0.104	TPD = −0.0192 PR +3.1318
Left 3, 4	0.344	TPD = 0.079 PR +1.2421
Left 5, 6, 7	0.1	TPD = 0.014 PR +2.4651
Left 8	0.44	TPD = −0.0835 PR +3.2377

### Correlation between diabetic foot two-point dicrimination and diabetic foot power ratio values – subjects without sensation

The above correlation has been calculated for each area in ‘without-sensation’ subjects and presented in Table 6. The corresponding regression equation was obtained by analysis of data of nine diabetic subjects in all specified areas of the foot sole. It is clear from the Table 6 that all the areas do not have any correlation between PR and TPD values. Therefore, higher PR value of foot sole does not give rise to higher values of TPD.

**Table 6:** Coefficient of correlation (r) between two-point discrimination and Power ratio (PR) values and the corresponding regression equations in different foot areas of the foot in diabetic subjects without sensation

Foot areas	Correlation coefficient (r)	Regression equation
Right 1, 2	0.154	TPD = −0.0499 PR +3.86
Right 3, 4	0.33	TPD = 0.0354 PR +1.671
Right 5, 6, 7	0.134	TPD = 0.011 PR +2.331
Right 8	0.104	TPD = 0.0157 PR +2.466
Left 1, 2	0.0002	TPD = 3E−055 PR +2.736
Left 3, 4	0.45	TPD = 0.1033 PR +1.089
Left 5, 6, 7	0.55	TPD = 0.0649 PR +1.524
Left 8	0.6	TPD = −0.1322 PR +3.806

## Discussion

While it was the bias of the investigators at the inception of the study that a higher TPD would be found related to increased skin hardness or increased PR, no such relationship was found. This is similar to the observation by Dellon *et al,*[13] showing the independence of shore values and TPD in fingers. This independence may be related to the unique and intimate physical juxtaposition of the Merkel cell-neurite complex to the intermediate ridge at the dermal/epidermal junction.[21] The slowly adapting fiber/receptor system that transmits the perception of pressure may function independently of skin compliance because the force is directly transmitted from skin surface to the nerve fiber/receptor.[22] Patil *et al,* have observed that the shore values and PR have high positive correlation in foot areas; pressure distribution as measured by the PR may be related to compliance as measured by the shore value. Therefore, the reason for the independence of TPD and shore values could be the same for the independence of TPD and PR.

The precise physiological mechanisms that sub serve TPD itself remain poorly elucidated,[21,23] but may be said to be independent of skin compliance and pressure distribution. This independence is probably due to the cortical processing required for TPD and needs further investigation.

Although SWMF is a widely used clinical tool to measure the sensory loss, we did not find any particular difference in the TPD values of DM subjects with and without sensation of 10 g monofilaments. Thus, there appears to be no particular additional value in measuring loss of protection with monofilaments in comparison to those that assesses the TPD. Similarly, the correlation tables shown above do not differ much with or without sensation.

It should be noted that our study is restricted to subjects clinically screened for peripheral artery occlusive disease (PAOD). There were no subjects with clinical vasculopathy. In the presence of maldistribution of blood supply, however, we do not know if these correlations are still valid. As Rendell *et al,*[24] stressed, the maldistribution between nutritional and thermoregulatory skin blood flow in the toes of diabetic patients could be directly related to the development of ulcers in the feet and the increase in TPD values. Also, it is to be investigated whether these correlations are specific for DM subjects or applicable to other disease conditions as well.

In this paper, we have studied only the correlations of TPD with shore values and PR. The correlations of other modalities of touch such as vibration detection threshold (VDT), cold detection threshold (CDT), warm detection threshold (WDT) and heat pain onset threshold (HPO) with the shore values and PR needs to be investigated.

## Conclusion

We have evaluated the correlation of different measures of sensory loss such as TPD, shore values and PR in both foot areas of DM subjects. We found that there is no correlation between TPD values measured using esthesiometer and shore values measured using shoremeter in the feet and no correlation between TPD and PR values measured using PPG in the feet. As shore values and PR were found to be highly positively correlated in other studies, TPD could represent different property of the foot compared to the other measures. The mechanism of this independence is not well understood and more investigation is required studying the relationships of the quickly adapting receptor system's perception of vibration and skin hardness and pressure distribution. The SWMF (10 g) is found not to provide any additional value in measuring loss of sensation in comparison to TPD. The correlations could help us in avoiding duplication of clinical tests and reduce overall healthcare cost.
